# A New Stopping Criterion for Rasch Trees Based on the Mantel–Haenszel Effect Size Measure for Differential Item Functioning

**DOI:** 10.1177/00131644221077135

**Published:** 2022-02-28

**Authors:** Mirka Henninger, Rudolf Debelak, Carolin Strobl

**Affiliations:** 1 University of Zurich, Switzerland

**Keywords:** differential item functioning, effect size, item response theory, Mantel–Haenszel odds ratio, Rasch tree

## Abstract

To detect differential item functioning (DIF), Rasch trees search for optimal splitpoints in covariates and identify subgroups of respondents in a data-driven way. To determine whether and in which covariate a split should be performed, Rasch trees use statistical significance tests. Consequently, Rasch trees are more likely to label small DIF effects as significant in larger samples. This leads to larger trees, which split the sample into more subgroups. What would be more desirable is an approach that is driven more by effect size rather than sample size. In order to achieve this, we suggest to implement an additional stopping criterion: the popular Educational Testing Service (ETS) classification scheme based on the Mantel–Haenszel odds ratio. This criterion helps us to evaluate whether a split in a Rasch tree is based on a substantial or an ignorable difference in item parameters, and it allows the Rasch tree to stop growing when DIF between the identified subgroups is small. Furthermore, it supports identifying DIF items and quantifying DIF effect sizes in each split. Based on simulation results, we conclude that the Mantel–Haenszel effect size further reduces unnecessary splits in Rasch trees under the null hypothesis, or when the sample size is large but DIF effects are negligible. To make the stopping criterion easy-to-use for applied researchers, we have implemented the procedure in the statistical software R. Finally, we discuss how DIF effects between different nodes in a Rasch tree can be interpreted and emphasize the importance of purification strategies for the Mantel–Haenszel procedure on tree stopping and DIF item classification.

## Introduction

Standardized educational and psychological tests are widespread tools to measure skills, aptitudes, or educational outcomes. When test-takers with the same ability but different characteristics, such as language, ethnicity, or gender, have a different probability of giving a correct response to an item, this item shows *differential item functioning* (DIF). When a test consists of a substantial amount of items showing DIF favoring one or more subgroups, it can affect test fairness (Camilli, 2006). Therefore, a variety of statistical methods to detect DIF have been proposed in order to identify DIF items.

Using the Rasch model, the probability that test-taker *n* gives a correct response ( $X_{\mathrm{ni}}=1$ ) to item *i* can be described in terms of a logistic function of the ability $\theta_{n}$ of test-taker *n* and the difficulty $\beta_{i}$ of item *i*:

$$P(X_{\mathrm{ni}}=1)=\frac{\exp(\theta_{n}-\beta_{i})}{1+\exp(\theta_{n}-\beta_{i})}$$

**Figure 1.**
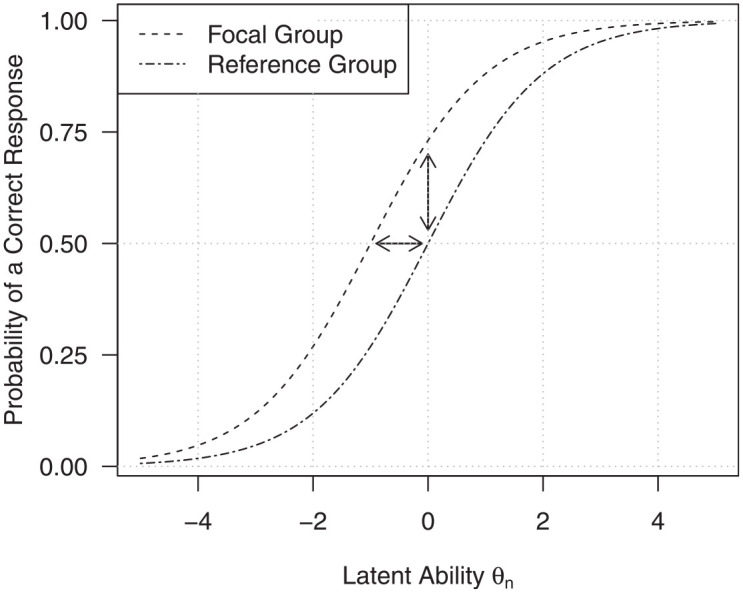
Item Characteristic Curves for an Item With Differential Item Functioning for Two Groups of Test-Takers.

The Rasch model assumes equal item difficulty parameters for all test-takers. However, in case of DIF, an item is not equally difficult for test-takers with the same ability level, but coming from different subgroups *g*. As an example, an item assessing math skills with an English task description might be more difficult for test-takers whose native language is not English than for English native speakers. As a consequence, the difficulty of this item ( $\beta_{i}$ ) is not equal across these subgroups, but subgroup specific ( $\beta_{\mathrm{ig}}$ ). It is typical in DIF analyses that these subgroups are binary, and they are typically named the focal and the reference group, respectively ( g∈{F,R} ). Figure 1 shows two exemplary *Item Characteristic Curves* (ICCs) for two subgroups of respondents. The ICCs illustrate the probability of giving a correct response on a given item as a function of the latent ability $\theta_{n}$ . On one hand, we can see that DIF is present because the item is not equally difficult for test-takers from the two subgroups, and the curves are shifted on the horizontal axis by the item difficulty difference (as is illustrated by the horizontal arrow). On the other hand, we can see that for an exemplary test-taker with latent ability $\theta_{n}=0$ it is more likely to give a correct response in case that such a test-taker belongs to the focal group compared with the reference group (as is illustrated by the vertical arrow; e.g., Steinberg & Thissen, 2006). As recommended by Steinberg and Thissen (2006), we will focus on DIF detection methods based on the horizontal difference, that is the difference in item difficulty between the reference and the focal group, rather than the vertical difference, that is the difference in response proportions. The former group of DIF detection methods aims at quantifying by which amount the ICC of the focal group is shifted horizontally compared with the ICC of the reference group.

The groups to be compared with respect to DIF are typically specified a priori based on available covariates. As most of the approaches use comparisons between a low number of groups (such as gender or a diagnosis), continuous or categorical variables with several categories, such as age or language, need to be dichotomized or discretized, for example, based on the median, before these standard procedures can be applied (e.g., Holland & Thayer, 1986, see also Strobl et al., 2015 for a discussion).

However, it is not always apparent how to choose a valid cutpoint in continuous and categorical variables, and an a priori discretization of covariates may not always lead to subgroups that are optimal for DIF detection. As an alternative, the Rasch tree method (Strobl et al., 2015) can be used to identify previously unknown DIF groups in a data-driven way. As Rasch trees find the optimal cutpoint in continuous or categorical covariates, they have a larger power to detect DIF compared with, for example, Andersen’s likelihood ratio test when the true cutpoint is not actually in the position that was specified a priori (see Strobl et al., 2015, for more details).

At the same time, Rasch trees also have some caveats: Since a statistical significance test is used to determine whether and where a split is performed, Rasch trees are more likely to detect small item parameter differences in larger samples, which are common in international large-scale assessments such as the Programme for International Student Assessment (PISA) or National Assessment of Educational Progress (NAEP). Thus, Rasch trees tend to show more splits, grow larger, and identify more subgroups in larger samples due to greater statistical power. However, small DIF effects may not always be relevant in practice. Therefore, an approach that is driven more by effect size and less by sample size would be desirable.

Furthermore, Rasch trees conduct a *global* invariance test to identify relevant covariates and optimal cutpoints. This means that they do not assess DIF in a particular item. Rather, they find the covariates and optimal cutpoints that identify those subgroups between which one or more of the item parameters differ. Hence, Rasch trees select covariates and optimal cutpoints, but they do not allow to automatically identify individual items with DIF or to quantify the magnitude of DIF for individual items. In addition, the more splits are performed, the larger the trees grow, and the more subgroups are identified. This makes it more difficult for users to identify the relevant covariates and DIF items, and therewith to interpret DIF effects substantively.

In this article, we propose a new stopping procedure for Rasch trees that is based on a sample size independent effect size measure. Such an effect size measure can be used to stop the tree from growing when differences in item parameters between the identified subgroups are minor in size. Furthermore, applied researchers are provided a pragmatic, explorative procedure not only to identify relevant covariates and their cutpoints via Rasch trees but also to identify DIF items and quantify the DIF effects for each split via the effect size measure.

### Detecting DIF Through Rasch Trees

The Rasch tree method (Strobl et al., 2015) is based on a model-based recursive partitioning approach that detects differences in model parameters (in our case item difficulties of an educational or psychological test) between subgroups of test-takers that are identified in a data-driven way based on available covariates. Herein, we briefly summarize the Rasch tree procedure using an empirical data example (for further details on the method please, see Strobl et al., 2015). We use the statistical programming environment R (R Core Team, 2020) together with the package psychotree (Strobl et al., 2015), which is based on the general algorithm for recursive partitioning of the package partykit (Hothorn & Zeileis, 2015).

**Figure 2.**
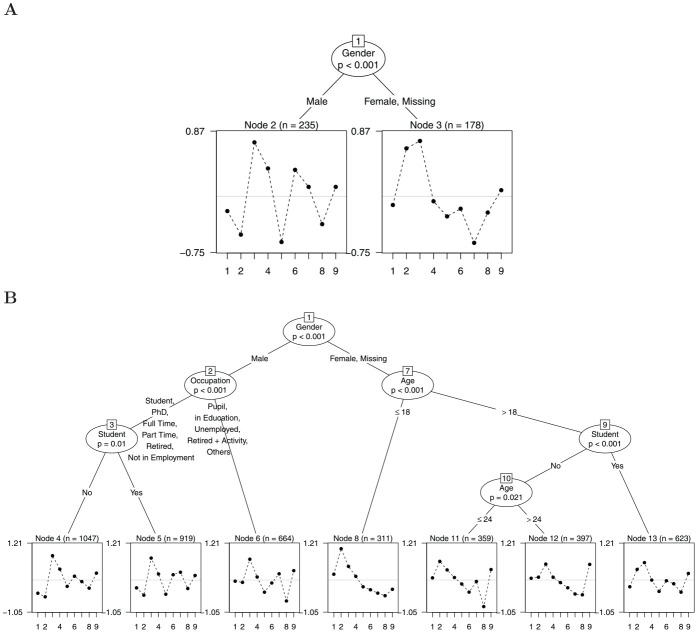
Example of Two Rasch Trees Based on the Nine Natural Science Items of a General Knowledge Quiz *Note.* Panel A is based on a random sample of N=500 test-takers, Panel B is based on a random sample of N=5,000 test-takers. End nodes display the item difficulty profiles in the identified subsamples with item number on the x-axis and item difficulty on the y-axis.

We demonstrate the method by means of data from an online general knowledge quiz conducted by a German weekly news magazine (Trepte & Verbeet, 2010). For illustrative purposes, we selected only the nine natural science items and randomly drew two samples of test-takers ( N=500 and N=5,000 ). Item responses to the nine items were analyzed in terms of item parameter differences with respect to the covariates “Age,”“Gender,”“Student,” and “Occupation” using the Rasch tree method. Figure 2 shows a fitted Rasch tree for the small and the large sample.

In Panel A using a sample size of N=500 , the identified subgroups are male test-takers compared with female test-takers and test-takers who did not indicate their gender.^1^ The estimated item difficulty profiles for each subsample are displayed in the end nodes of the tree (Nodes 2 and 3) with item number on the x-axis and item difficulty on the y-axis. As the Rasch tree has identified subgroups for which item parameters differ, we can conclude that DIF is present in the analyzed data. From a visual inspection, we see that item parameters differ between the subgroups such that, for example, relative to the other items, Item 2 (“What is ultrasound not used for?—Radio”) seems to be less difficult for male test-takers, whereas Item 4 (“What is also termed Trisomy 21?—Down Syndrom”) seems to be less difficult for female or missing gender test-takers.

In Panel B of Figure 2, we can see the fitted Rasch tree for sample size N=5,000 . Compared with Panel A, we see that much more splits have been detected for the available covariates. These additional splits result in seven identified subgroups in which the gender subgroups are additionally partitioned into subgroups based on the covariates “Age,”“Student,” and “Occupation.”

Until now, two types of stopping criteria could be used when fitting a Rasch tree (Strobl et al., 2015). First, each of the splits performed by the Rasch tree is based on a statistical significance test that evaluates the instability in item difficulty parameters with respect to available covariates. Tree growing is stopped when the item parameter instability test is nonsignificant given a certain α-level. Second, tree growing is stopped when a minimum sample size in a node is reached. So, as sample size increases, the power to detect even small DIF effects increases and a minimum sample size in a node is reached later in the tree growing process. Hence, both stopping criteria are less likely to be met for larger samples and the Rasch tree is more likely to split even when DIF effects are small.

**Figure 3.**
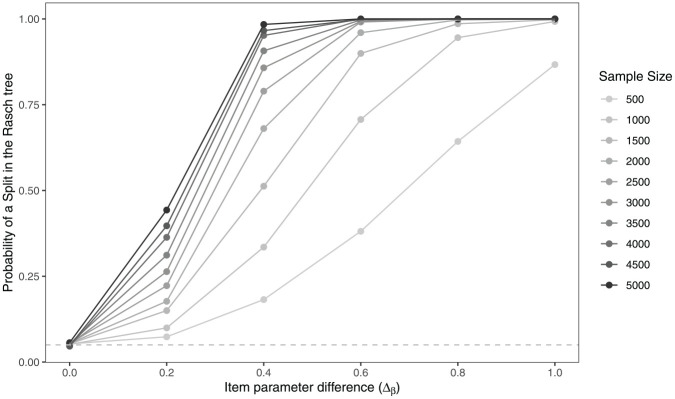
Type I Error Rate (at $\Delta_{\beta }=0$ ) and Power (for Higher Effect Sizes) of Rasch Trees to Detect Differential Item Functioning (DIF) in One Item Based on a Dichotomous Covariate as a Function of the Item Parameter Difference ( $\Delta_{\beta }$ ) and Sample Size Based on *R* = 2,000 Simulation Replications.

To illustrate the statistical power of Rasch trees for different sample sizes, Figure 3 displays the probability that a Rasch tree performs a split on a dichotomous covariate as a function of sample size and the item parameter difference ( $\Delta_{\beta }=\beta_{\mathrm{iR}}-\beta_{\mathrm{iF}}$ ). While the Rasch tree holds the Type I error rate of α=.05 when no DIF is present ( $\Delta_{\beta }=0$ ), like for any significance test, we find a pattern where the probability of a split in the tree increases with increasing effect size, but also with increasing sample size. Depending on sample size, the probability ranges between $18{\rm %}$ and $98{\rm %}$ when $\Delta_{\beta }=0.4$ , a negligible to medium-sized DIF effect (Paek & Fukuhara, 2015; Phillips & Holland, 1987). Therefore, in particular for small item parameter differences and larger samples, an additional stopping criterion is needed. Such a stopping criterion can validate a split in the tree in terms of its effect size and stop the tree from growing when the effect size is small. Therefore, we extend the stopping criteria of Rasch trees by adding a commonly used effect size quantification approach for DIF based on the popular Educational Testing Service (ETS) classification scheme of the Mantel–Haenszel (MH) odds ratio in the Δ-metric.

### MH Effect Size Measure

We will briefly review the MH effect size measure together with the ETS classification scheme that we will use to quantify uniform DIF. Uniform DIF means that one group is uniformly more likely to give a correct response than the other group. This implies that slope or discrimination parameters are equal between the groups (i.e., parallel ICCs as in Figure 1), and only the difficulty parameters differ between the groups.

The MH odds ratio (Mantel, 1963; Mantel & Haenszel, 1959) is a popular measure for quantifying uniform DIF (Holland & Thayer, 1986). It expresses the strength of a relation between the group membership and the response in a test, conditional on the ability level (Holland & Thayer, 1985, 1986). It is commonly reported in the Δ-metric, which has been proposed by the ETS and is a linear transformation of the logarithm of the MH odds ratio (Holland & Thayer, 1985, see below).

To calculate the MH odds ratio, test-takers are divided into *j* ability levels. These ability levels are called the *matching criterion*. As a proxy for the ability level, often the sum score of the test is used. Hence, it is implicitly assumed that the sum score is a valid surrogate for the unobserved ability and that DIF is balanced, meaning that advantages due to DIF in certain items cancel out disadvantages due to DIF in other items (we will revisit this aspect further below). Then, the reference (“R”) and focal (“F”) groups matched on their sum scores are compared with respect to the proportion of correct (“1”) and incorrect (“0”) responses on a given item. Table 1 depicts the contingency table that is used to calculate the MH odds ratio. Herein, $A_{j}$ and $C_{j}$ reflect the number of correct responses, while $B_{j}$ and $D_{j}$ reflect the number of incorrect responses, and $m_{\mathrm{Rj}}$ and $m_{\mathrm{Fj}}$ reflect the total number of responses in the reference and focal group for ability level *j*, respectively. The total number of correct and incorrect responses are reflected by $m_{1j}$ and $m_{0j}$ , whereas $T_{j}$ is the total number of test-takers with the jth ability level.

**Table 1.** 2×2 Contingency Table for a Given Item and Ability Level *j*.

Group	Correct (1)	Incorrect (0)	Total
Reference (R)	$A_{j}$	$B_{j}$	$m_{\mathrm{Rj}}$
Focal (F)	$C_{j}$	$D_{j}$	$m_{\mathrm{Fj}}$
Total	$m_{1j}$	$m_{0j}$	$T_{j}$

The MH statistic is computed as the odds ratio over all *j* ability levels (Holland & Thayer, 1986; Magis et al., 2010; Steinberg & Thissen, 2006)

(1) $$\alpha_{\mathrm{MH}}=\frac{\sum_{j}A_{j}D_{j}/T_{j}}{\sum_{j}B_{j}C_{j}/T_{j}}.$$

The logarithm of the odds ratio $\lambda_{\mathrm{MH}}=\log(\alpha_{\mathrm{MH}})$ is asymptotically normally distributed (Magis et al., 2010). Values around 0 indicate that the item is DIF-free, while larger positive or negative values indicate that DIF in favor of the reference or focal group is present, respectively. Holland and Thayer (1985) proposed a linear transformation to a Δ-metric^2^ so that

(2) $$\Delta_{\mathrm{MH}}=-2.35\cdot \lambda_{\mathrm{MH}}$$

Again, when item difficulties are equal in the focal and reference group $\Delta_{\mathrm{MH}}=0$ , while $\left|\Delta_{\mathrm{MH}}\right|$ increases with increasing difficulties between the focal and reference group. This $\Delta_{\mathrm{MH}}$ effect size for DIF has been classified into negligible (“A”), medium (“B”), and large (“C”) DIF effects using a classification scheme by ETS (Paek & Holland, 2015; Zwick, 2012) that is displayed in Table 2. The classification rule includes (a) an evaluation of the absolute size of $\Delta_{\mathrm{MH}}$ together with (b) a statistical significance test to evaluate whether $\Delta_{\mathrm{MH}}$ is not significantly different from 0 (class “A”) or significantly larger than 1 (class “C”), which depends on both effect size and sample size. In consequence, in classifying $\Delta_{\mathrm{MH}}$ , its absolute effect size is most decisive. The statistical significance test comes into play when assessing DIF in small samples. Here, evaluating the significance in addition to the absolute effect size can avoid classifications in classes “B” and “C” when the differences between the focal and reference group have occurred by chance.

**Table 2.** ETS Classification Scheme for the Mantel–Haenszel Odds Ratio in the Δ-Metric ( $\Delta_{\mathrm{MH}}$ ).

Class	Interpretation	Classification rule
A	Negligible DIF	$|\Delta_{\mathrm{MH}}|\leq 1$ or not significantly different from 0
B	Medium DIF	Neither A nor C
C	Large DIF	$|\Delta_{\mathrm{MH}}|\geq 1.5$ and significantly larger than 1

*Note.* ETS = educational testing service; DIF = differential item functioning; α=.05 .

The significance test can be realized using Shervish’s *p* value formula (see Paek & Holland, 2015) testing $|\Delta_{\mathrm{MH}}|>\tau$ with

(2) $$\rho_{\tau }({\hat{\Delta }}_{\mathrm{MH}})=\Phi \left(\frac{-\tau -|{\hat{\Delta }}_{\mathrm{MH}}|}{s}\right)+\Phi \left(\frac{\tau -|{\hat{\Delta }}_{\mathrm{MH}}|}{s}\right)$$

With τ=0 when a classification in category A is tested, while τ=1 for classification in category C, and $s=2.35\sqrt{\sigma^{2}({\hat{\lambda }}_{\mathrm{MH}})}$ (Paek & Fukuhara, 2015; Phillips & Holland, 1987).

### Extending Rasch Trees by the MH Effect Size

Steinberg and Thissen (2006) demonstrate that when the Rasch model holds and uniform DIF is to be measured, the difference between item difficulty parameters (horizontal arrow in Figure 1) is actually equivalent to $\Delta_{\mathrm{MH}}$ (see Holland & Thayer, 1985; Roussos et al., 1999 see Equation 2), such that

(2) $$\Delta_{\mathrm{MH}}\equiv -2.35\cdot (\beta_{\mathrm{iR}}-\beta_{\mathrm{iF}})$$

Hence, up to a scaling constant that places the value on the ETS Δ-scale, $\Delta_{\mathrm{MH}}$ is equivalent to the difference in the item parameters between the focal and reference groups when DIF is uniform (Steinberg & Thissen, 2006). This makes $\Delta_{\mathrm{MH}}$ a suitable measure for evaluating DIF effect sizes in Rasch trees. Furthermore, the $\Delta_{\mathrm{MH}}$ method has certain advantages when it comes to integrating an effect size into the Rasch tree procedure. First, as the calculation of $\Delta_{\mathrm{MH}}$ solely requires information on group membership and item responses, it can be implemented in a computationally inexpensive way (see Equations 1 and 2; see also Rogers & Swaminathan, 1993; Vaughn & Wang, 2010) and does not require to estimate a parametric model. Second, $\Delta_{\mathrm{MH}}$ is a popular and widely used DIF effect size measure. Hence, using $\Delta_{\mathrm{MH}}$ as a stopping criterion in Rasch trees is appealing, as researchers working with DIF are already acquainted with the meaning of this effect size measure and in communicating the results to their readers. Therefore, the widely accepted classification scheme of $\Delta_{\mathrm{MH}}$ into the A, B, and C categories will serve as a basis for a new Rasch tree stopping criterion such that $\Delta_{\mathrm{MH}}$ must fall at least in category “B” for at least one item of the test to validate the split. Hence, splitting is stopped when DIF effects for all items are categorized as “A.”

#### Purification of the Matching Criterion

Many parametric DIF detection methods rely on DIF-free items, so-called *anchor items*, that are used to fix a common scale for comparing item difficulty parameters between the focal and reference group (see, for example, Glas & Verhelst, 1995; Kopf et al., 2015a, 2015b). Finding optimal anchor items has been under intensive evaluation and discussion, as DIF test results can be affected when anchor items are not DIF-free (so-called contamination, see, for example, Finch, 2005; Kopf et al., 2015b; Wang & Su, 2004a).

Anchor selection is not only important in parametric DIF approaches but also for the calculation of the $\Delta_{\mathrm{MH}}$ statistic. When calculating $\Delta_{\mathrm{MH}}$ as in Equations 1 and 2, the sum score is only a good matching criterion when the test contains no items with DIF, or when DIF is balanced between the reference and focal group, such that differences in response probabilities due to DIF cancel each other out across items. If none of these unlikely assumptions is met, there is a risk that the matching criterion is contaminated by DIF items, similar to the use of an equal-mean-difficulty or all-other anchor method in the area of anchor selection (e.g., Wang et al., 2012).

There are several strategies to *purify* the matching criterion, and the most popular are two-step (Clauser et al., 1993) and iterative purification (Kok et al., 1985; van der Flier et al., 1984). Both are similar to an iterative backward anchor, where DIF items are excluded from the anchor (here the sum score) in an iterative procedure (French & Maller, 2007; Wang et al., 2012). Using a two-step purification procedure on the $\Delta_{\mathrm{MH}}$ statistic, the sum score is used as a matching criterion for the initial DIF analysis. In a second step, a new sum score without the items that were identified to have DIF is computed for the final DIF analysis (Kok et al., 1985). When using iterative purification, this process is repeated until two runs yield the same DIF items (Kok et al., 1985; van der Flier et al., 1984) or until a maximum value of iterations is reached (Socha et al., 2015). Please note that while items that have previously been identified as DIF items are excluded from the matching criterion when $\Delta_{\mathrm{MH}}$ is calculated, the item under investigation *always* contributes to the calculation of the matching criterion in this approach (Holland & Thayer, 1986; Raju et al., 1989; Zwick, 1990). Another noteworthy characteristic of the purified $\Delta_{\mathrm{MH}}$ odds ratio is that when DIF is evaluated with respect to different groups, the purified matching criterion may differ. For example, one subset of items may be selected as the matching criterion when evaluating DIF between younger and older people, but another subset of items may be selected when evaluating DIF between language groups from the same data set.

There is a large number of studies comparing one or both purification strategies to a nonpurified approach (e.g., Clauser et al., 1993; Crane et al., 2006; DeMars, 2020; Fidalgo et al., 2000; French & Maller, 2007; Guilera et al., 2013; Hidalgo-Montesinos & Gómez-Benito, 2003; Khalid, 2011; Kwak et al., 1998; Navas-Ara & Gómez-Benito, 2002; Rogers & Swaminathan, 1993; Socha et al., 2015; Teresi, 2006; Wang et al., 2009; Wang & Su, 2004a, 2004b). Both, the two-step as well as the iterative purification procedure have been shown to have higher DIF detection rates (Clauser et al., 1993; Fidalgo et al., 2000; Guilera et al., 2013), while iterative purification has the largest advantage over the other approaches when a large proportion of items have DIF (Fidalgo et al., 2000; French & Maller, 2007; Navas-Ara & Gómez-Benito, 2002). Therefore, we firmly recommend the use of a purification strategy (two-step or iterative) when using the $\Delta_{\mathrm{MH}}$ method as a stopping criterion in Rasch trees.

The remainder of the article is structured as follows. In a simulation study, we show that the inclusion of $\Delta_{\mathrm{MH}}$ in Rasch trees further reduces Type I error rates and splits for small DIF effects. Finally, we illustrate and discuss implications of the stopping procedure based on an empirical example. We also briefly describe how we have implemented the new $\Delta_{\mathrm{MH}}$ procedure into the Rasch tree method in the software environment R in Appendix A.

## Simulation Studies

In order to assess whether the effect size-based stopping procedure is a valuable tool in Rasch trees, we tested its effects on tree stopping and item classification using simulation studies. We assessed a no-DIF condition ( $H_{0}$ ), where the true DIF effect size was zero, and DIF conditions ( $H_{1}$ ) with increasing DIF effect sizes. Both conditions, $H_{0}$ and $H_{1}$ , were each assessed in two data settings: (a) a simple setting with only one dichotomous covariate, which is presented in detail in the main article and (b) a more complex setting with one dichotomous and one continuous covariate.

The simple setting (a) allows us to demonstrate the effects of $\Delta_{\mathrm{MH}}$ in Rasch trees in a lucid, interpretable setting. At the same time, the main advantage of Rasch trees comes with identifying optimal cutpoints in categorical or continuous covariates. Therefore, we additionally provide simulation results for the more complex but also more realistic setting (b) as a sanity check. The simulation results of the more complex setting are comparable to those in the simple setting, while the results’ presentation becomes quite complex as additional splits in the Rasch trees are possible. Hence, presenting the results for data setting (a) in the main text would unnecessarily lengthen the results’ presentation, and thus details on the simulation setting (b) can be found in Appendix B.

For all simulations, we used the software R with the packages partykit and psychotree (Hothorn & Zeileis, 2015; Strobl et al., 2015) to fit the Rasch trees, the difR package (Magis et al., 2010) to calculate the MH odds ratio, additional R code for purifying the $\Delta_{\mathrm{MH}}$ effect size and using it as a stopping criterion in Rasch trees (cf. Appendix A), and ggplot2 (Wickham, 2016) for plotting.

### Simulation Setup for $\Delta_{\mathrm{MH}}=0$

When generating data without DIF ( $H_{0}$ ), we varied sample size ( N∈(500,1000,2000,5000) ) and test length ( I∈(20,40) ). Person parameters were drawn from a standard normal distribution N(0,1) , whereas item parameters were drawn from a uniform distribution U(−2,2) and centered. In data setting (a), a dichotomous covariate was sampled from a binomial distribution with $50{\rm %}$ of the values in each of the two categories. Using this setup, we generated binary data from a Rasch model that was DIF-free ( $\Delta_{\mathrm{MH}}=0$ , i.e., item difficulty parameters were independent of the covariate).

The generated Rasch data were analyzed with the Rasch tree method using the dichotomous covariate as a potential splitting variable. In case, the Rasch tree detected a split and partitioned the data into two subgroups, we calculated the $\Delta_{\mathrm{MH}}$ effect size measure for each item using the two subgroups as the focal and reference group. We calculated three $\Delta_{\mathrm{MH}}$ effect sizes, using no purification, two-step purification, and iterative purification to compare the purification strategies. Given an α -level of α=.05 for a split in the Rasch trees and given that we aimed to evaluate approximately 2,500 split Rasch trees in each condition, we realized R=50,000 replications under $H_{0}$ .

As a criterion for stopping, we defined that all items must have been classified in category “A” of the $\Delta_{\mathrm{MH}}$ procedure (i.e., negligible DIF). In case that a split in the Rasch tree occurred, we assessed how often the Rasch tree would have been stopped by our new $\Delta_{\mathrm{MH}}$ -based stopping rule and classified items into the ETS categories “A,”“B,” and “C.” Please remember that under $H_{0}$ , independent of sample size, a Rasch tree with an α -level of α=.05 is expected to split in $5{\rm %}$ of the replications. However, as is the case for any significance test, in smaller samples, only large differences between the subgroups that occur by chance will lead to a significant result. In contrast, in larger samples already small coincidental differences between the subgroups will lead to a significant result. Therefore, we expect the effect size-based stopping criterion to have a negligible impact on tree stopping when sample size is small. However, when sample size is large, we expect that the effect size based stopping criterion can detect splits that were based on negligible differences between the subgroups and avoid these superfluous splits. As will be shown in the following section, our simulation studies support both expectations.

### Results for Tree Stopping and Item Classification for ${\Delta _{MH}} = 0$

**Figure 4.**
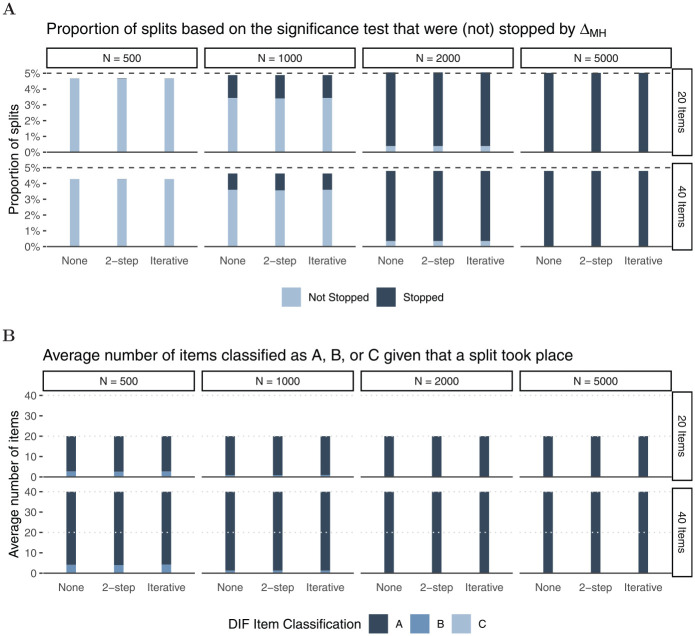
Simulation Results for $H_{0}$ *Note.* Panel A: Proportions of splits based on the significance test (light and dark parts of the bars together; dark parts of the bars: Splits that were stopped by the $\Delta_{\mathrm{MH}}$ rule). The dashed line reflects the nominal alpha rate in the Rasch tree ( α=.05 ); Panel B: Average number of items that were classified as “A,”“B,” or “C” given that a split took place.

In Panel A of Figure 4, the light and dark parts of the bars together show the proportion of splits. As expected, we find that Rasch trees perform a split in approximately $5{\rm %}$ of the replications, hence holding the Type I error rate independent of sample size or number of items. The dark part of the bars indicate the proportion of splits that were stopped through $\Delta_{\mathrm{MH}}$ . In order for a split to be performed under $H_{0}$ based on the significance test, the coincidental difference in item parameters must be substantially larger for smaller than for large samples.^3^ Thus, as expected, the stopping procedure has negligible impact in smaller samples ( N=500 ), while we find a substantial proportion of splits being stopped by the new $\Delta_{\mathrm{MH}}$ rule in larger samples. For example, for *N* = 2,000 more than $90{\rm %}$ of trees with splits are stopped by the new stopping rule. This further lowers false positive DIF detection rates.

Panel B of Figure 4 shows how many items were classified as “A,”“B,” or “C” for all performed splits in Rasch trees (complete bars in Panel A) averaged across simulation replications. For instance, in the first panel ( N=500 , I=20 ), we see that in trees with splits out of 20 items only a negligible number of items ( ≤0.12 ) were classified as “C,” a small number of items ( ≤2.56 ) were classified as “B,” while the majority of items ( ≥17.33 ) were classified as “A.” As expected, larger samples lead to more accurate DIF classifications with more items being classified as “A” (negligible DIF) under $H_{0}$ . In addition, and in line with previous studies, we find minor effects of number of items and purification strategy (none, two-step, or iterative purification; see also Wang, 2004) on item classification when no DIF effects are present in the data-generating process. The results for data-generating process (b), that included an additional continuous covariate, are largely comparable to those presented herein (see Appendix B for more details).

We conclude that under $H_{0}$ the Rasch tree holds its $5{\rm %}$ Type I error rate as expected, but the Type I error rate can further be reduced by means of the $\Delta_{\mathrm{MH}}$ stopping rule. In particular for larger samples where the differences between the subgroups in the generated data are negligible, $\Delta_{\mathrm{MH}}$ seems to be a very effective additional stopping procedure.

### Simulation Setup for $\Delta_{\mathrm{MH}}\in (0.5,1,...,2.5)$

For generating data containing DIF, we varied the proportion of DIF items ($5{\rm %}$ or $20{\rm %}$) and DIF effect size ( $\Delta_{\mathrm{MH}}\in (0.5,1,...,2.5)$ in addition to sample size and test length.

These effect sizes cover DIF effects classified as negligible ( ≤1 ), medium ( $1<\Delta_{\mathrm{MH}}<1.5$ ), and large ( ≥1.5 , see Table 2) and are equivalent to item parameter differences of $\Delta_{\beta }\in (0.21,0.43,0.64,0.85,1.06)$ . This choice of DIF effect sizes provides a realistic setting in which we cover small to large DIF effects (see also Chang et al., 2015; Pohl et al., 2021, for comparable choices of the range of DIF effects).

Using this setup, we generated binary data from a Rasch model that contained DIF through an item parameter difference between the focal and reference group according to $\Delta_{\beta }$ for DIF items. As the proportion of DIF items was varied ($5{\rm %}$ or $20{\rm %}$), 1 or 4 items contained DIF when the test length was 20 items, whereas 2 or 8 items contained DIF when the test length was 40 items. In data setting, (a) DIF was operationalized as an item parameter difference between the focal and reference group so that all items with DIF were easier for one of the two groups (nonbalanced DIF; see Appendix B for data setting (b)).

We realized a lower number of replications ( R=2,500 ) than under $H_{0}$ as we expected a larger proportion of splits under $H_{1}$ .

### Results for Tree Stopping and Item Classification for $\Delta_{\mathrm{MH}}\in (0.5,1,...,2.5)$

**Figure 5.**
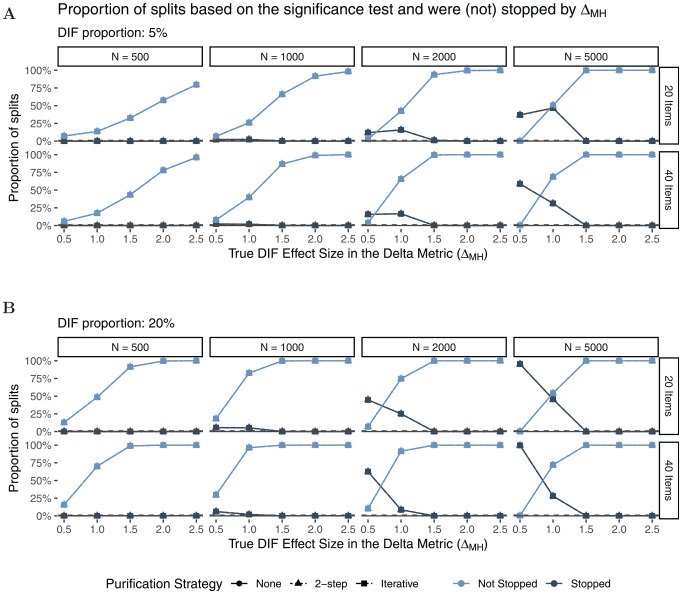
Proportion of Splits Based on the Significance Test (Light and Dark Shapes) That Were not Stopped (Light) or Were Stopped (Dark) by the $\Delta_{\mathrm{MH}}$ Rule Under $H_{1}$ .

Figure 5 shows the proportion of splits that were stopped by the $\Delta_{\mathrm{MH}}$ rule. The light-colored lines indicate the proportion of splits that were not stopped by $\Delta_{\mathrm{MH}}$ , whereas the dark-colored lines indicate the proportion of splits that were stopped. As expected, the larger the effect size (x-axis), the more likely was the Rasch tree to perform a split overall. We can also see that the proportion of Rasch trees that were stopped by the $\Delta_{\mathrm{MH}}$ rule becomes larger as sample size increases. This was expected as splits for small effect sizes have a higher probability to be found significant in larger rather than smaller samples. Rasch trees are mainly stopped by the new $\Delta_{\mathrm{MH}}$ rule for small sizes of DIF ( $\Delta_{\mathrm{MH}}\leq 1.0$ ), whereas no trees are stopped by the new rule when data with large DIF effects ( $\Delta_{\mathrm{MH}}\geq 1.5$ ) were generated. In addition, stopping based on the $\Delta_{\mathrm{MH}}$ rule is more likely to occur in shorter tests, as the stopping criterion that all items are classified in category “A” is less likely to be met as test length increases. This is in line with a conservative strategy, where we do not want splits for negligible DIF, but also do not want to miss non-negligible DIF effects. Finally, the purification strategy does not seem to have a substantial influence on stopping (but see the results for classification further below for differences between purification strategies).

**Figure 6.**
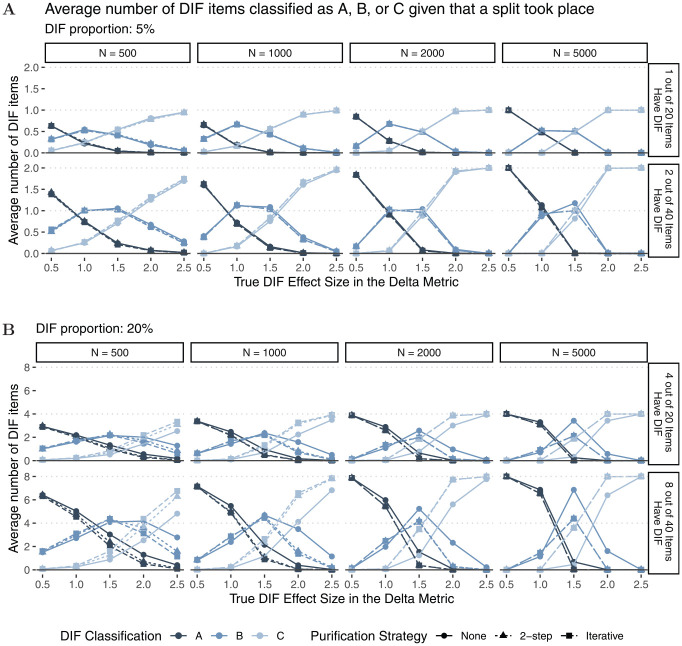
Average Number of DIF Items That Have Been Classified as A, B, or C Given That a Split Took Place *Note.* Panel A: DIF proportion: 5% ; Panel B: DIF proportion: 20% . DIF = differential item functioning.

Figure 6 shows the average number of items that were classified in category “A,”“B,” or “C” for all performed splits (light- and dark-colored lines in Figure 5). As expected, neither sample size nor test length seems to have substantial effects on DIF classification. In line with Fidalgo et al. (2000) and French and Maller (2007), and Navas-Ara and Gómez-Benito (2002), we find small differences between purification strategies for low DIF proportions (Panel A), but larger differences between purification strategies for higher DIF proportions (Panel B) and larger DIF effect sizes. Based on the steeper slope of the classification curves for larger DIF effect sizes, we conclude that two-step as well as iterative purification seem to better discriminate between DIF and non-DIF items when a substantial amount of DIF is present. Overall, we find that item classification matches true DIF effect sizes as items with larger true DIF effect sizes are classified in higher DIF categories. The results for data-generating process (b), which included an additional continuous covariate, are largely comparable to those presented here (see Appendix B for more details).

In summary, the simulations support the use of $\Delta_{\mathrm{MH}}$ as a stopping procedure in Rasch trees, as it can help to avoid false positive splits and splits based on negligible DIF in Rasch trees. In addition, it can be used to assess the magnitude of DIF effects. Under $H_{0}$ , the inclusion of effect sizes further reduces the proportion of split trees, in particular for samples with N≥2000 . When DIF is present, the use of the effect size avoids splits in Rasch trees when the sample size is large but DIF effects are negligible ( $\Delta_{\mathrm{MH}}\leq 1$ ). While the purification strategy seems to have minor effects on stopping, it does have substantial effects on DIF item classification (i.e., the interpretation of DIF effects), with minor differences between the two-step and the iterative approach.

One may argue that more settings with additional or other types of covariates must be covered via simulations in order to validate the $\Delta_{\mathrm{MH}}$ procedure for its use in empirical data. At this point, it is noteworthy that the Rasch tree is based on a recursive procedure. This means that each split in a Rasch tree is performed successively, and each split is performed only if the preceding split took place. The $\Delta_{\mathrm{MH}}$ effect size, in turn, is only computed given a split in the Rasch tree. Hence, the Rasch tree follows a closed testing procedure which ensures that multiple splits do not lead to an inflated Type I error rate and that the postulated significance level holds for the entire Rasch tree (Hochberg & Tamhane, 1987; Marcus et al., 1976; Strobl et al., 2015). We have tested the combination of the Rasch tree with $\Delta_{\mathrm{MH}}$ in a setting using one dichotomous, but also in a setting with a continuous and a dichotomous covariate without detecting violations with regard to Type I error, and it is not to be expected that additional covariates would change this behavior.

## Illustration Using Empirical Data

**Figure 7.**
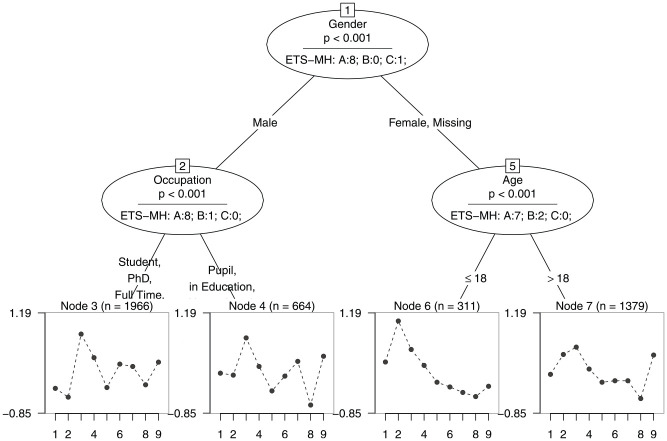
Example of a Rasch Tree Using the $\Delta_{\mathrm{MH}}$ Stopping Rule (to be Compared With Panel B in Figure 2) With a Display of ETS Classification of $\Delta_{\mathrm{MH}}$ in the Oval Nodes Representing the Splits *Note.* End nodes display the item difficulty profiles in the identified subsamples with item number on the x-axis and item difficulty on the y-axis. ETS = Educational Testing Service.

We reanalyzed the data from the general knowledge quiz used in the introduction, now using the new $\Delta_{\mathrm{MH}}$ stopping rule.^4^ Herein, we used the same random sample of N=5,000 test-takers that resulted in the Rasch tree in Panel B in Figure 2. The resulting tree can be seen in Figure 7. We see that compared with the tree in Panel B in Figure 2, additional splits on the covariates “Student” and “Age” have been avoided. As a consequence, the tree has four end nodes compared with the seven end nodes obtained without the new stopping procedure.

**Figure 8.**
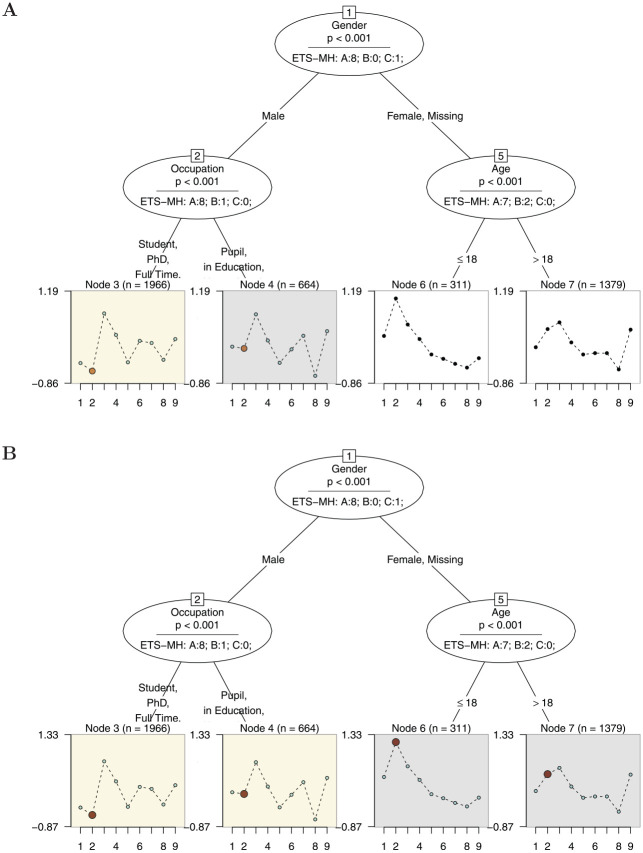
Panel A: Example of a Rasch Tree Using the $\Delta_{\mathrm{MH}}$ Stopping Rule With End Nodes Colored by Oval Node 2 (Split in the Covariate “Occupation”); Panel B: End Nodes are Colored by Oval Node 1 (Split in the Covariate “Gender”) *Note.* End nodes display the item difficulty profiles in the identified subsamples with item number on the x-axis and item difficulty on the y-axis.

In addition, we extended the information of the Rasch tree by displaying the number of items classified in categories “A,”“B,” or “C” in each inner node. Furthermore, the item parameters displayed in the end nodes can be colored by the category in which they were classified as is shown in Figure 8. For instance, in Panel A in Figure 8, we have colored the items based on the oval for Node 2 representing the split in the covariate “Occupation” (resulting in Nodes 3 and 4). We can see that with respect to “Occupation” eight items have been classified as “A,” while one item has been classified as “B” and no item as “C.” From the item parameter profiles in Nodes 3 and 4, we can directly see that, via $\Delta_{\mathrm{MH}}$ , Item 2 has been classified in category “B” (medium DIF).

Furthermore, end nodes linked to the oval that was selected for comparison are automatically colored in the background. This procedure is further illuminated using the Rasch tree displayed in Panel B. Here, the end nodes are colored by the oval for Node 1 (split in the covariate “Gender”) where eight items showed DIF in category “A,” and one item showed DIF in category “C.” Again, the item classified as “C” (Item 2) is colored in the end nodes linked to the oval for Node 1 (Nodes 3, 4, 6, and 7). In addition, the background color directly indicates that DIF was based on a split in Node 1. Hence, Nodes 3 and 4 have to be compared with Nodes 6 and 7 for interpreting the DIF effect and are colored differently in the background. From the item parameter profiles in the end node panels, we can conclude that compared with the other items, Item 2 is less difficult for male test-takers than for female test-takers and test-takers who did not indicate their gender.

For facilitating this kind of comparison, our extension of the Rasch tree also uses those items that have been identified as DIF-free by the $\Delta_{\mathrm{MH}}$ procedure as anchor items for the respective comparison of two (groups of) end nodes. For example, when comparing Node 3 versus Node 4 in Panel A in Figure 8 (or Nodes 3 and 4 vs. Nodes 6 and 7 in Panel B), the item parameters are displayed using the DIF-free items (i.e., those items colored in light blue) from this particular comparison as anchor items. In this particular simple example, the effect of the anchoring is almost imperceptibly small, but this approach ensures that in more complex situation an appropriate anchor is used for each comparison. For all nodes that are not used in the current comparison, a sum-zero constraint across all items is used for displaying the item parameters, as is the default in the psychotree package.

## General Discussion

In this article, we propose to use an effect size measure of DIF to support the evaluation of whether a split in a Rasch tree is based on a meaningful difference in item parameters. For this purpose, we have implemented the popular MH odds ratio using the ETS classification scheme as an additional stopping criterion in the Rasch tree method. This effect size-based stopping criterion provides additional information on DIF effect sizes given that the Rasch tree has selected a covariate and identified an optimal cutpoint based on a statistical significance test. The effect size measure provides additional information that can be used to define a stopping criterion and avoid superfluous splits, but it can also be used to quantify the size of the DIF effect. The simulation studies have shown that the effect size stopping criterion further reduces Type I error rates and avoids splits in Rasch trees when the sample size is large, but DIF effects are negligible. Furthermore, the additional stopping criterion also allows users to identify the most relevant DIF items in each split. Besides the conceptual integration of the effect size measure into the Rasch tree procedure, we also implemented this extension together with an improved anchoring strategy of the item parameter profiles in R in order to make it available for applied researchers in an easy-to-use way (see Appendix A).

We defined the stopping criterion such that all items must be categorized in category “A” to prevent a split in the Rasch tree. Unsurprisingly, our simulation studies showed that stopping becomes slightly more unlikely for larger tests. This is because the criterion that all items are categorized as “A” is more unlikely when the number of items increases. We believe that this observation does not jeopardize the use of $\Delta_{\mathrm{MH}}$ as a stopping procedure in practical applications. Our simulation results have shown that the impact of test length on stopping is observable, but negligible in size (cf. Panel A in Figures 4 and 10), and of course, the Rasch tree holds its Type I error rate independent of whether an additional stopping procedure is used (Strobl et al., 2015). Of course, users of the new $\Delta_{\mathrm{MH}}$ stopping procedure can also formulate their own, potentially more liberal or strict, stopping rules such as that stopping should occur when all item are classified in categories “A” or “B.”

A related aspect is that the probability of finding at least one DIF item under $H_{0}$ is larger for longer tests. Correction measures, such as the Bonferroni procedure (see, for example, Simes, 1986) or the Benjamini–Hochberg procedure to control the false discovery rate (Benjamini & Hochberg, 1995, see also Thissen et al., 2002), are available for the MH $\chi^{2}$ test, but not for the $\Delta_{\mathrm{MH}}$ effect size with the ETS classification scheme. For the $\Delta_{\mathrm{MH}}$ effect size with categories “A,”“B,” and “C,” users should remain aware that due to item-wise DIF evaluation, the probability of incorrectly detecting DIF in at least one item increases with test length (Fidalgo et al., 2000; Zwick, 2012). A possible avenue for future research might, therefore, be the development of alternative effect sizes for item-wise DIF that explicitly take the overall test length into account.

It is important to note that as a characteristic of recursive partitioning, the Rasch tree algorithm does not search over all possible partitions. As a consequence, it does not necessarily find the globally optimal solution. We found an example of this characteristic in the empirical data used (Figures 2 and 7). Here, only items with negligible DIF (“A”) were found in the split in the covariate “Student” (Node 9 in Panel B, Figure 2). However, when classifying the items in the follow-up node (Node 10 in Panel B, Figure 2), two items showed medium DIF (“B”). This highlights that in certain situations (e.g., a split with all items showing at most negligible DIF occurs, but DIF is present in a follow-up node), the recursive partitioning algorithm in combination with stopping procedures may risk to miss certain DIF effects and DIF subgroups.

It is noteworthy that our extension of Rasch trees in terms of the effect size can be used in several ways. On one hand, the effect size–based stopping can be used to avoid superfluous splits, as we have demonstrated in the simulation studies and in the illustration with empirical data. This makes the Rasch tree easier to interpret. On the other hand, evaluating $\Delta_{\mathrm{MH}}$ for each item in each split provides additional information with respect to the magnitude of DIF effects, even when it is not used as a stopping procedure. Hence, if users fear to miss any DIF effect, they could refrain from using $\Delta_{\mathrm{MH}}$ as a stopping procedure, but nevertheless rely on $\Delta_{\mathrm{MH}}$ as a measure of DIF magnitude.

The Rasch tree method with the new stopping rule is an exploratory procedure to detect important covariates and split points as well as to identify DIF items in a data-driven way. It may be feared that as a data-driven approach, the partitioning in the Rasch tree might not be stable across different data samples. To respond to this concern, the stablelearner package (Philipp et al., 2018) provides descriptive and graphical analyses for assessing the stability of variable and cutpoint selection based on resampling (see Philipp et al., 2016; Strobl et al., 2021, for tutorials). Besides examining present data with regard to DIF effects, the Rasch tree procedure could also be used to generate hypotheses, for instance about the causal link of DIF covariates and items. Of course, new hypotheses that are generated based on the results of an exploratory procedure like the Rasch tree method would require new data to be thoroughly tested.

While the MH odds ratio in the Δ-metric can be used to quantify DIF for dichotomous items, it cannot be applied to polytomous items, which are more commonly used for measuring personality traits or attitudes. As DIF in polytomous items can be explored in the recursive partitioning framework using partial credit trees (Komboz et al., 2018), we are currently working on extending the partial credit tree framework by an effect size-based stopping procedure. For instance, an adaptation of the gamma coefficient for partial credit models may be a fruitful candidate as, similar to $\Delta_{\mathrm{MH}}$ , it is a popular measure to classify DIF items in three categories (“A,”“B,” and “C”; Bjorner et al., 1998; Kreiner, 1987).

We conclude that by classifying whether differences in item parameters are ignorable or not, the effect size measure facilitates identifying items that differ between subgroups and quantifying the magnitude of these differences in each split of the Rasch tree.

## A. Software Implementation

Two R packages are in use when a Rasch tree is fit: the package partykit provides the recursive partitioning algorithm used to identify relevant covariates and cutpoints, whereas the package psychotree integrates the Rasch model into the recursive partitioning algorithm.

The partitioning algorithm works as follows (Strobl et al., 2015):

- for a given sample, fit a Rasch model
- test whether there exists a covariate for which item parameters are instable and test for significance
- if there is a significant instability, find the optimal cutpoint and perform a split
- repeat the procedure until no further significant instability is found or a minimum sample size is reached.

We are in contact with the package authors in order to implement the $\Delta_{\mathrm{MH}}$ stopping procedure into the partykit and psychotree packages in the next version(s). Until then, a set of helper functions can be installed from Github.^5^ Please note that the functionality is under current development, is likely to change or be implemented in a different way in the future, and builds on package versions partykit 1.2 and psychotree0.15 using R 4.0.2.

In the following, we provide some details about the implementation for the interested reader. We have added the option for a user-defined stopping function (stopfun) to the control arguments of the mob function in the package partykit. This control option stopfun allows to hand over a user-defined function to the tree growing algorithm. Using the item response data and an indicator of the subgroup assignment of the current split, splitting is stopped when stopfun evaluates to TRUE, while splitting is continued otherwise. Therewith, stopfun allows the user to check whether an already performed split in the tree was valid in a “post check.” The effect size–based stopping is implemented in this way, because to calculate the $\Delta_{\mathrm{MH}}$ statistic the allocation of test-takers to subgroups must already have taken place.

In the current Github implementation, the control option stopfun can directly be handed over via the raschtree function using the functionality of the psychotree package. We provide a helper function stopfun_mantelhaenszel that takes additional arguments, such as the type of purification strategy and the stopping criterion. As an example, using the following code to fit a Rasch tree, it would be stopped if all items in a split were classified as “A” between two subgroups (see Table 2):

rasch_tree_fit <- raschtree (resp ~ age + language, data = dat, stopfun = stopfun_mantelhaenszel( purification = “iterative”, stopcrit = “A”,)

When purification = “2step,” the statistic is computed and purification is performed on the statistic. When purification = “iterative,” the maximum number of purification steps is set to the number of items in the test. When purification = “none,” the statistic is computed, but no purification is performed.

While the recursive partitioning algorithm implemented in partykit can be used for many different statistical models, such as polytomous models (Komboz et al., 2018), linear or logistic regression (Zeileis et al., 2008), Bradley Terry models (Strobl et al., 2011), or multinomial processing tree models (Wickelmaier & Zeileis, 2018), the $\Delta_{\mathrm{MH}}$ statistic is a stopping criterion specifically for Rasch models. Therefore, the information about the effect size and ETS classification are not saved in the generic tree object during the estimation process but are added to the info section of the Rasch tree object afterward using the add_mantelhaenszel function, from where it can be accessed via $info$mantelhaenszel.

rasch_tree_MH <- add_mantelhaenszel(rasch_tree_fit, purification = “iterative”) rasch_tree_MH $info$mantelhaenszel

When the plotting method is applied to the MH-based Rasch tree object, the ETS classification for each split can be shown together with the information in the inner node (see Figure 7), and the item parameters in the end nodes can be colored based on the DIF classification (“A,”“B,”“C”) of a split in the Rasch tree (see Figure 8). The coloring of item parameters occurs for all end nodes that are linked to the inner node (the difference in item parameters can be seen in end nodes on the left side versus end nodes on the right side of the inner node). As outlined in the main article, item parameters are anchored using the DIF-free items for the respective comparison, while in all other end nodes, the item parameters are anchored using a sum-zero constraint across all items.

plot(rasch_tree_MH, show_classification = TRUE, color_by_node = 2)

## B. Detailed Simulation Results for an Additional Continuous Covariate

Here, we present the results of the simulation study that uses a more complex data setting (b) where in addition to a dichotomous covariate, a continuous covariate served as a splitting candidate. We used the same simulation setup as presented in the main article with respect to sample size, test length, DIF proportion, and DIF effect size. The only difference was that, in addition to the binary covariate, a value for a continuous covariate was randomly sampled from a uniform distribution U~(1,100) for each respondent. Under $H_{0}$ ( $\Delta_{\mathrm{MH}}$ =0 ), neither the dichotomous nor the continuous covariate were related to item difficulty parameters. Under $H_{1}$ ( $\Delta_{\mathrm{MH}}$ ∈(0.5,1,...,2.5) ), respondents with values above 40 on the continuous covariate had an advantage in responding to DIF items according to the DIF size condition, while no DIF effect was added for the dichotomous variable.

### B1. Results for Tree Stopping and Item Classification for $\Delta_{\mathrm{MH}}=0$

**Figure 9.**
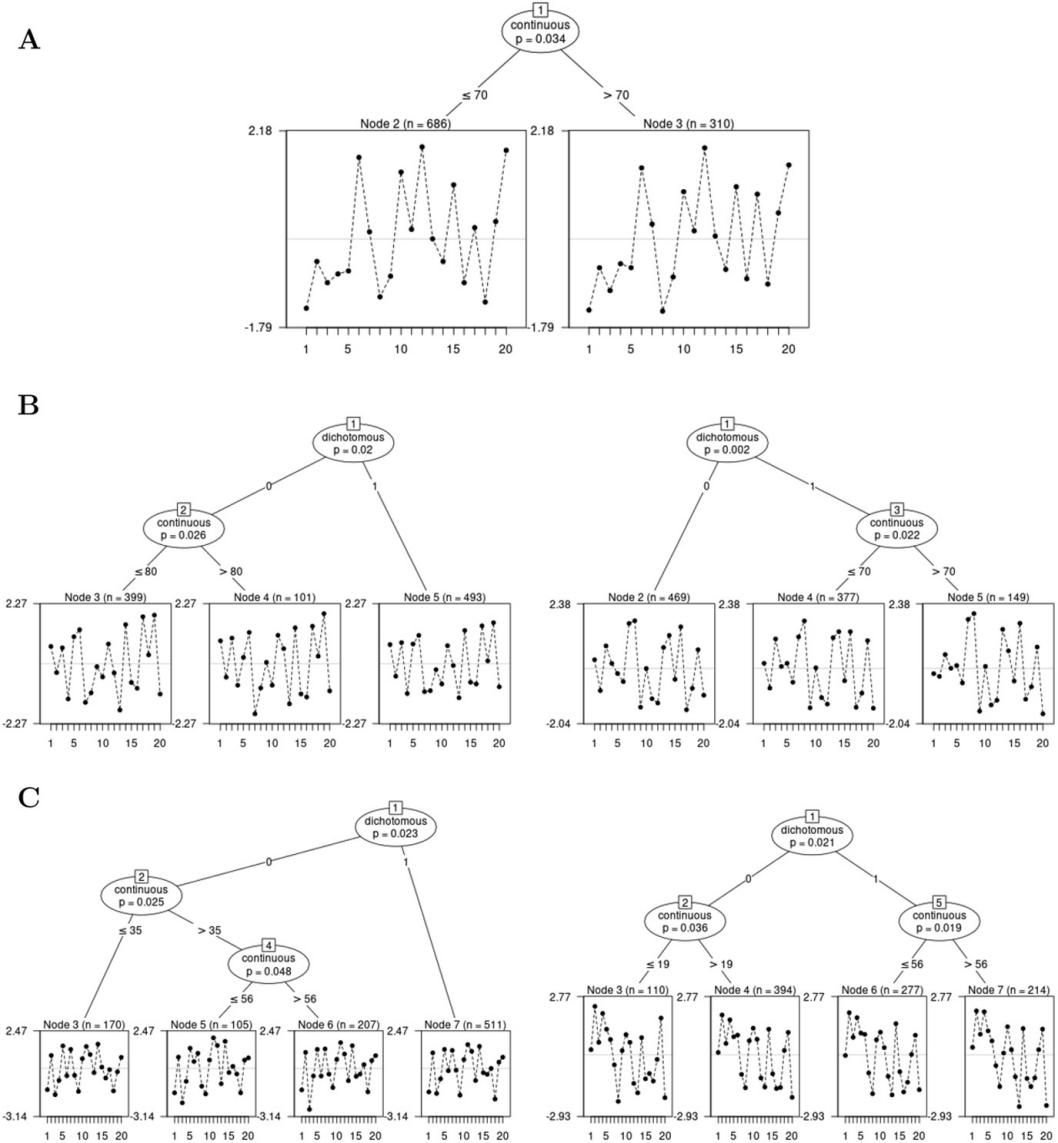
Exemplary Rasch Trees From Simulation 2 for *N* = 1,000 and I=20 ; Panel A: One Split in Node 1, Panel B: Two Splits, One in Node 1 and One in Node 2 or Node 3, Panel C: Exemplary Rasch Trees With More Than Two Splits.

As, in addition to a dichotomous covariate, a continuous covariate was added to the data-generating process, multiple splits in the tree were possible. We realized R=50,000 replication in each of the eight conditions (four sample size conditions, two test lengths) resulting in 400,000 fitted Rasch trees overall. Figure 9 illustrates some of the Rasch trees that are possible in this simulation setup. Mirroring the overall $5{\rm %}$ Type I error rate under $H_{0}$ , we found a tree with one split (Panel A) in $4.19{\rm %}$ of replications, a tree with two splits (Panel B) in $ 0.28{\rm %}$ of replications. Trees with more than two splits (such as in Panel C) were found in only 57 out of the 400,000 cases overall. As these trees with more than two splits occurred so seldomly but complicate the presention of the results, we only present results for trees with one or two splits (as, for example, in Panels A and B in Figure 9) in the following.

**Figure 10.**
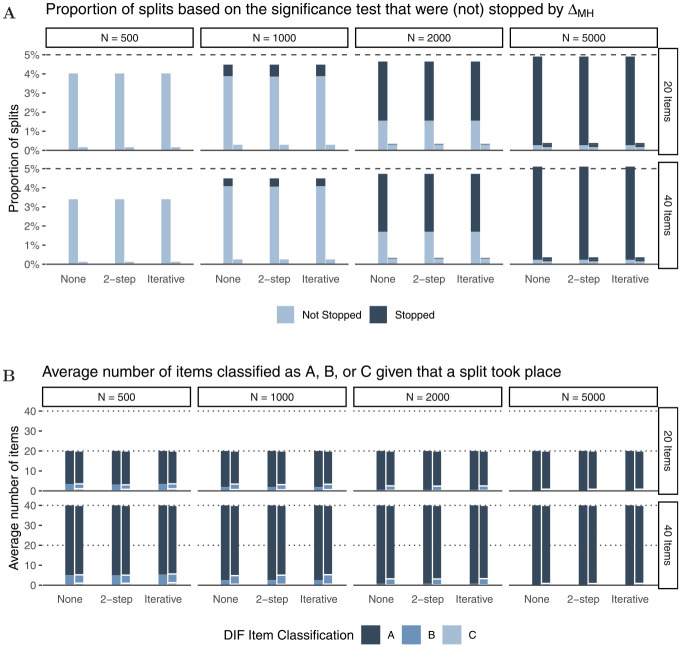
Simulation Results for $H_{0}$ *Note.* Panel A: Proportions splits based on the significance test (light and dark parts of the bars together); dark parts of the bars: splits that were stopped by the $\Delta_{\mathrm{MH}}$ rule. The dashed line reflects the alpha error rate in the Rasch tree ( α=.05 ); Panel B: Average number of DIF items that have been classified as A, B, or C given that a split took place; the left bar reflects the first node, the right bar reflects the second node in both panels. DIF = differential item functioning.

In Panel A of Figure 10, the complete bars (light and dark bars jointly) show the proportion of splits with each left bar reflecting splits in the first node, and each second (smaller) bar reflecting splits in a second node. Similar to the data-generating process (a), the stopping procedure (dark bars) has less prominent effects in smaller samples ( N=500 ) and more substantial effects in larger samples with most of the trees being stopped in the N=5,000 condition. In the rare cases that a second split occurred, stopping was performed very rarely, in particular for N≤2,000 . Compared with splits in the dichotomous covariate (data-generating process (a)), the Rasch tree is slightly more conservative under $H_{0}$ in smaller samples when a continuous covariate is used for the split. The reduced Type I error rate is to be expected due to the underlying test statistic for continuous covariates in Rasch trees on top of a Bonferroni correction taking place when more than one covariate serves as a candidate for a split (see also Strobl et al., 2015). As the proportion of splits in the first node minimally exceeds the $5{\rm %}$ boundary for *N* = 5,000 and 40 items, we also inspected the distribution of *p* values in the Rasch tree method itself across replications. We found the *p* values to approximately follow the expected uniform distribution with negligible levels of skewness, in particular for the larger sample sizes.

Looking at item classification in Panel B of Figure 10, we find negligible effects of test length and purification strategy, similar to the results for data-generating process (a). Again, most items are classified in category “A,” with larger samples leading to more accurate DIF classification. Please note that Panel B displays the average number of items classified for given splits which, however, only occured in $ 0.28{\rm %}$ of replications for the second node.

### Results for Tree Stopping and Item Classification for $\Delta_{\mathrm{MH}}\in (0.5,1,...,2.5)$

For every split, we classified items in categories “A,”“B,” and “C.” Similar to the simulation under $H_{0}$ , we excluded trees with more than two inner nodes, as these only occurred in 66 (three inner nodes), 752 (four inner nodes), or 5 (five inner nodes) of the replications compared with 79,266 for one or 9,646 for two inner nodes (out of 100,000 trees that were fit in the 4×2×5 design with R=2,500 replications). In order to test the validity of the $\Delta_{\mathrm{MH}}$ stopping procedure with a continuous variable, we examined the splits that were based on the continuous covariate and excluded splits based on the dichotomous covariate from the results’ presentation. As DIF proportion, test length, and purification strategy had minor effects on the splits conducted and stopped, we only present results based on tests with 20 items and a DIF proportion of $20{\rm %}$ to make the results’ presentation more concise.

**Figure 11.**
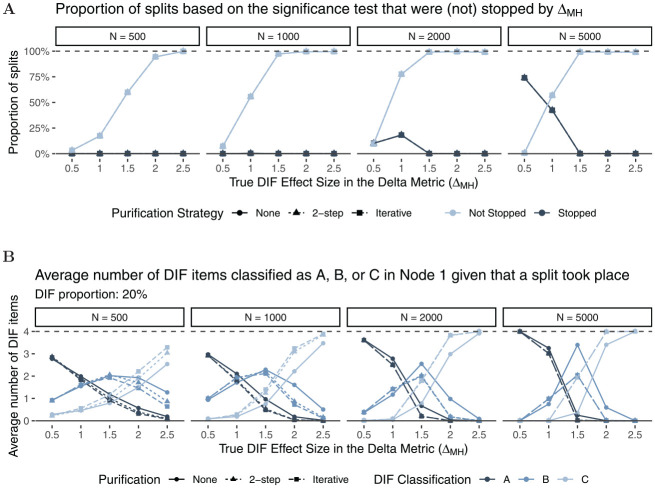
Panel A: Proportion of Splits of the Continuous Covariate in Node 1 Based on the Significance Test (Light and Dark Shapes) but Were not Stopped (Light) or Stopped (Dark) by the $\Delta_{\mathrm{MH}}$ Rule Under $H_{1}$ . Panel B: Average number of DIF items that have been classified as A, B, or C given that a split of the continuous covariate in Node 1 took place. DIF = differential item functioning.

Panel A in Figure 11 depicts the proportion of splits that were stopped based on the continuous covariate as a function of sample size (columns) and DIF effect size (x-axis) for Node 1 (Panel A). Here, we see a pattern similar to the one for the simple data-generating process (a), where more splits occurred for larger effect sizes, and also more splits based on small DIF effect sizes ( $\Delta_{\mathrm{MH}}\leq 1$ ) were stopped.

Panel B in Figure 11 shows the average number of DIF items classified as “A,”“B,” or “C” based on the continuous covariate in Node 1 as a function of sample size (columns), DIF effect size (x-axis) and purification strategy (solid, dotted, and dashed lines) for 20 item tests and a DIF proportion of $20{\rm %}$. Here, a similar pattern as for data-generating process (a) emerged such that the larger the sample size the more the classification reflects true DIF effect sizes. The curves’ slopes (i.e., the discrimination between DIF effect size categories) become steeper as sample size increases and for two-step or iterative purification compared with nonpurified $\Delta_{\mathrm{MH}}$ .

**Figure 12.**
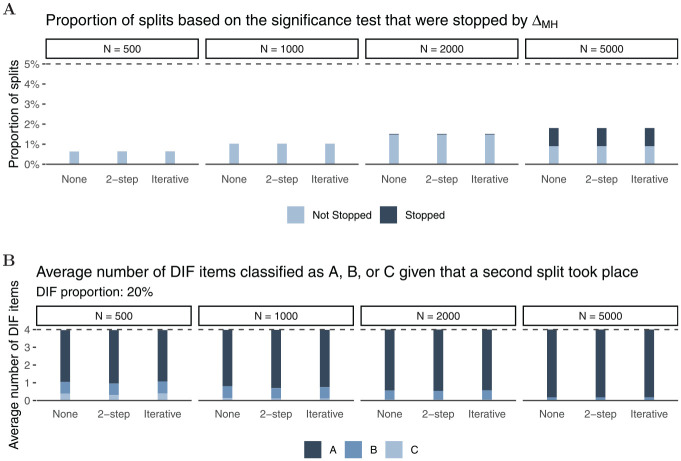
Panel A: Proportions of Splits of the Continuous Covariate in a Second Node Based on on the Significance Test (Light and Dark Shapes). Panel B: Average number of DIF items that have been classified as A, B, or C given a split of the continuous covariate in a second node. DIF = differential item functioning.

Figure 12 shows the proportion of splits as well as the DIF classification for a split in a second node (Panel A). As the continuous covariate was generated such that a perfect cut-off at value 40 separated the focal from the reference group, we expected a small amount of splits, as DIF induced in the data-generating process should have been captured in Node 1. Indeed, very few splits occurred in a second node ($ \lt 1.8{\rm %}$) and a substantial proportion of splits was stopped when sample size was large ( N=5,000 ). Similarly, we can see that the majority of items are classified in category “A,” indicating that even if a second split occurred, the risk of classifying items in categories “B” or “C,” hence assigning medium or large DIF effect sizes to items, remains small (Panel B).

## Notes

1. Please note that the variable gender was binary coded (female/male) in the original dataset, while $11.4{\rm %}$ of test-takers did not indicate their gender. We decided to code missing as an additional category of the gender variable for the Rasch tree analysis.

2. Holland and Thayer (1985) linearly transformed $\lambda_{\mathrm{MH}}$ so it would have the same interpretation as the ETS item difficulty, which has already been measured in a Δ-metric (Holland & Thayer, 1986).

3. Besides the results presented in the figures, this is also reflected in the absolute differences between item parameters, which become smaller (i.e., more accurate since the data were generated under $H_{0}$ ) in larger samples (e.g., for N=500 : $|\bar{\Delta \hat{\beta }}|=0.31$ , for N=5,000 : $|\bar{\Delta \hat{\beta }}|=0.10$ ).

4. As a reminder: In the “Introduction,” we used data from an online general knowledge quiz (Trepte & Verbeet, 2010), selected the nine natural science items, and randomly drew two samples of test-takers ( N=500 and *N* = 5,000). We assessed DIF with respect to the covariates “Age,”“Gender,”“Student,” and “Occupation.” In the following, we will focus only on the tree for the larger sample *N* = 5,000, because the Rasch tree is harder to interpret due to its numerous splits. Furthermore, as demonstrated in the simulation studies (Panel A in Figure 4 and 10), $\Delta_{\mathrm{MH}}$ has negligible to no effects with regard to stopping splits in Rasch trees for smaller sample sizes, such as N=500 .

5.
https://github.com/mirka-henninger/raschtreeMH

## References

[bibr1-00131644221077135] Benjamini Y. Hochberg Y. (1995). Controlling the false discovery rate: A practical and powerful approach to multiple testing. Journal of the Royal Statistical Society. Series B: Statistical Methodology, 57, 289–300. http://www.jstor.com/stable/2346101

[bibr2-00131644221077135] Bjorner J. B. Kreiner S. Ware J. E. Damsgaard M. T. Bech P. (1998). Differential item functioning in the Danish translation of the SF-36. Journal of Clinical Epidemiology, 51, 1189–1202. 10.1016/S0895-4356(98)00111-5 9817137

[bibr3-00131644221077135] Camilli G. (2006). Test fairness. In Brennan R. (Ed.), Educational measurement (pp. 221–256). ACE/Praeger Series on Higher Education.

[bibr4-00131644221077135] Chang Y. W. Huang W. K. Tsai R. C. (2015). DIF detection using multiple-group categorial CFA with minimum free baseline approach. Journal of Educational Measurement, 52, 181–199. 10.1111/jedm.12073

[bibr5-00131644221077135] Clauser B. E. Mazor K. M. Hambleton R. K. (1993). The effects of purification of the matching criterion on the identification of DIF using the MH procedure. Applied Measurement in Education, 6, 269–279. 10.1207/s15324818ame0604_2

[bibr6-00131644221077135] Crane P. K. Gibbons L. E. Jolley L. van Belle G. (2006). Differential item functioning analysis with ordinal logistic regression techniques: DIFdetect and difwithpar. Medical Care, 44, 115–123. 10.1097/01.mlr.0000245183.28384.ed 17060818

[bibr7-00131644221077135] DeMars C. E. (2020). Alignment as an alternative to anchor purification in DIF analyses. Structural Equation Modeling: A Multidisciplinary Journal, 27, 56–72. 10.1080/10705511.2019.1617151

[bibr8-00131644221077135] Fidalgo Á. M. Mellenbergh G. J. Muñiz J. (2000). Effects of amount of DIF, test length, and purification type on robustness and power of Mantel-Haenszel procedures. Methods of Psychological Research Online, 5, 43–53. https://citeseerx.ist.psu.edu/viewdoc/download?doi=10.1.1.586.7639&rep=rep1&type=pdf

[bibr9-00131644221077135] Finch H. (2005). The MIMIC model as a method for detecting DIF: Comparison with Mantel-Haenszel, SIBTEST, and the IRT likelihood ratio. Applied Psychological Measurement, 29, 278–295. 10.1177/0146621605275728

[bibr10-00131644221077135] French B. F. Maller S. J. (2007). Iterative purification and effect size use with logistic regression for differential item functioning detection. Educational and Psychological Measurement, 67, 373–393. 10.1177/0013164406294781

[bibr11-00131644221077135] Glas C. A. W. Verhelst N. D. (1995). Testing the Rasch model. In Fischer G. H. Molenaar I. W. (Eds.), Rasch models: Foundations, recent developments, and applications (pp. 69–95). Springer. 10.1007/978-1-4612-4230-7_5

[bibr12-00131644221077135] Guilera G. Gómez-Benito J. Hidalgo M. D. Sánchez-Meca J. (2013). Type I error and statistical power of the Mantel-Haenszel procedure for detecting DIF: A meta-analysis. Psychological Methods, 18, 553–571. 10.1037/a0034306 24127986

[bibr13-00131644221077135] Hidalgo-Montesinos M. D. Gómez-Benito J. (2003). Test purification and the evaluation of differential item functioning with multinomial logistic regression. European Journal of Psychological Assessment, 19, 1–11. 10.1027//1015-5759.19.1.1

[bibr14-00131644221077135] Hochberg Y. Tamhane A. (1987). Multiple comparison procedures. John Wiley & Sons.

[bibr15-00131644221077135] Holland P. W. Thayer D. T. (1985). An alternate definition of the ETS delta scale of item difficulty. Technical Report, 85, 1–8. https://onlinelibrary.wiley.com/doi/pdf/10.1002/j.2330-8516.1985.tb00128.x

[bibr16-00131644221077135] Holland P. W. Thayer D. T. (1986). Differential item functioning and the Mantel-Haenszel procedure. Program Statistics Research Technical Report No. 86-69, 1986, i–24. 10.1002/j.2330-8516.1986.tb00186.x

[bibr17-00131644221077135] Hothorn T. Zeileis A. (2015). partykit: A modular toolkit for recursive partitioning in R. Journal of Machine Learning Research, 16, 3905–3909. http://jmlr.org/papers/v16/hothorn15a.html

[bibr18-00131644221077135] Khalid M. N. (2011). The performance of Mantel-Haenszel procedures in the identification of DIF items. International Online Journal of Educational Sciences, 3, 435–447. http://www.ajindex.com/dosyalar/makale/acarindex-1423904378.pdf

[bibr19-00131644221077135] Kok F. G. Mellenbergh G. J. van der Flier H. (1985). Detecting experimentally induced item bias using the iterative logit method. Journal of Educational Measurement, 22, 295–303. 10.1111/j.1745-3984.1985.tb01066.x

[bibr20-00131644221077135] Komboz B. Strobl C. Zeileis A. (2018). Tree-based global model tests for polytomous Rasch models. Educational and Psychological Measurement, 78, 128–166. 10.1177/0013164416664394 29795950 PMC5965621

[bibr21-00131644221077135] Kopf J. Zeileis A. Strobl C. (2015a). A framework for anchor methods and an iterative forward approach for DIF detection. Applied Psychological Measurement, 39, 83–103. 10.1177/0146621614544195 29880995 PMC5978507

[bibr22-00131644221077135] Kopf J. Zeileis A. Strobl C. (2015b). Anchor selection strategies for DIF analysis: Review, assessment, and new approaches. Educational and Psychological Measurement, 75, 22–56. 10.1177/0013164414529792 29795811 PMC5965509

[bibr23-00131644221077135] Kreiner S. (1987). Analysis of multidimensional contingency tables by exact conditional tests: Techniques and strategies. Scandinavian Journal of Statistics, 14, 97–112. https://www.jstor.org/stable/4616054

[bibr24-00131644221077135] Kwak N. Dvenport E. C. Davison M. L. (1998). A comparative study of observed score approaches and purification procedures for detecting differential item functioning [Paper Presentation]. Paper presented at the Annual Meeting of the National Council on Measurement in Education, San Diego, CA, United States.

[bibr25-00131644221077135] Magis D. Béland S. Tuerlinckx F. De Boeck P. (2010). A general framework and an R package for the detection of dichotomous differential item functioning. Behavior Research Methods, 42, 847–862. 10.3758/BRM.42.3.847 20805607

[bibr26-00131644221077135] Mantel N. (1963). Chi-Square tests with one degree of freedom: Extensions of the Mantel-Haenszel procedure. Journal of the American Statistical Association, 58(303), 690–700. 10.1080/01621459.1963.10500879

[bibr27-00131644221077135] Mantel N. Haenszel W. (1959). Statistical aspects of the analysis of data from retrospective studies of disease. Journal of the National Cancer Institute, 22, 719–748. https://academic.oup.com/jnci/article-abstract/22/4/719/900746 13655060

[bibr28-00131644221077135] Marcus R. Peritz E. Gabriel K. (1976). Closed testing procedures with special reference to ordered analysis of variance. Biometrika, 63(3), 655–660. 10.1093/biomet/63.3.655

[bibr29-00131644221077135] Navas-Ara M. J. Gómez-Benito J. (2002). Effects of ability scale purification on the identification of DIF. European Journal of Psychological Assessment, 18, 9–15. 10.1027//1015-5759.18.1.9

[bibr30-00131644221077135] Paek I. Fukuhara H. (2015). Estimating a DIF decomposition model using a random-weights linear logistic test model approach. Behavior Research Methods, 47, 890–901. 25134667 10.3758/s13428-014-0512-9

[bibr31-00131644221077135] Paek I. Holland P. W. (2015). A note on statistical hypothesis testing based on log transformation of the Mantel-Haenszel common odds ratio for differential item functioning classification. Psychometrika, 80, 406–411. 10.1007/s11336-013-9394-5 24337958

[bibr32-00131644221077135] Philipp M. Rusch T. Hornik K. Strobl C. (2018). Measuring the stability of results from supervised statistical learning. Journal of Computational and Graphical Statistics, 27, 685–700. 10.1080/10618600.2018.1473779

[bibr33-00131644221077135] Philipp M. Zeileis A. Strobl C. (2016). A toolkit for stability assessment of tree-based learners. In Colubi A. Blanco A. Gatu C. (Eds.), Proceedings of COMPSTAT 2016—22nd International Conference on Computational Statistics (pp. 315–325). The International Statistical Institute/International Association for Statistical Computing. https://www.zeileis.org/papers/Philipp+Zeileis+Strobl-2016.pdf

[bibr34-00131644221077135] Phillips A. Holland P. W. (1987). Estimators of the variance of the Mantel-Haenszel log-odds-ratio estimate. Biometrics, 43, 425–431. 10.2307/2531824 2790113

[bibr35-00131644221077135] Pohl S. Schulze D. Stets E. (2021). Partial measurement invariance: Extending and evaluating the cluster approach for identifying anchor items. Applied Psychological Measurement, 45, 477–493. 10.1177/01466216211042809 34866708 PMC8640350

[bibr36-00131644221077135] R Core Team. (2020). R: A language and environment for statistical computing. https://www.r-project.org

[bibr37-00131644221077135] Raju N. S. Bode R. K. Larsen V. S. (1989). An empirical assessment of the Mantel-Haenszel statistic for studying differential item performance. Applied Measurement in Education, 2, 1–13. 10.1207/s15324818ame020L1

[bibr38-00131644221077135] Rogers H. J. Swaminathan H. (1993). A comparison of logistic regression and Mantel-Haenszel procedures for detecting Differential Item Functioning. Applied Psychological Measurement, 17, 105–116. 10.1177/014662169301700201

[bibr39-00131644221077135] Roussos L. A. Schnipke D. L. Pashley P. J. (1999). A generalized formula for the Mantel-Haenszel Differential Item Functioning parameter. Journal of Educational and Behavioral Statistics, 24, 293–322. 10.3102/10769986024003293

[bibr40-00131644221077135] Simes R. J. (1986). An improved Bonferroni procedure for multiple tests of significance. Biometrika, 73, 751–754. https://www.jstor.org/stable/pdf/2336545.pdf

[bibr41-00131644221077135] Socha A. DeMars C. E. Zilberberg A. Phan H. (2015). Differential item functioning detection with the Mantel-Hanszel procedures: The effects of matching types and other factors. International Journal of Testing, 15, 193–215. 10.1080/15305058.2014.984066

[bibr42-00131644221077135] Steinberg L. Thissen D. (2006). Using effect sizes for research reporting: Examples using item response theory to analyze differential item functioning. Psychological Methods, 11, 402–415. 10.1037/1082-989X.1L4.402 17154754

[bibr43-00131644221077135] Strobl C. Kopf J. Zeileis A. (2015). Rasch trees: A new method for detecting differential item functioning in the Rasch model. Psychometrika, 80, 289–316. 10.1007/s11336-013-9388-3 24352514

[bibr44-00131644221077135] Strobl C. Schneider L. Kopf J. Zeileis A. (2021). Using the raschtree function for detecting differential item functioning in the Rasch model. http://rsync.udc.es/CRAN/web/packages/psychotree/vignettes/raschtree.pdf 10.1007/s11336-013-9388-3 24352514

[bibr45-00131644221077135] Strobl C. Wickelmaier F. Zeileis A. (2011). Accounting for individual differences in Bradley-Terry models by means of recursive partitioning. Journal of Educational and Behavioral Statistics, 36, 135–153. 10.3102/1076998609359791

[bibr46-00131644221077135] Teresi J. A. (2006). Different approaches to differential item functioning in health applications: Advantages, disadvantages and some neglected topics. Medical Care, 44, 152–170. 10.1097/01.mlr.0000245142.74628.ab 17060822

[bibr47-00131644221077135] Thissen D. Steinberg L. Kuang D. (2002). Quick and easy implementation of the Benjamini-Hochberg procedure for controlling the false positive rate in multiple comparisons. Journal of Educational and Behavioral Statistics, 27, 77–83. 10.3102/10769986027001077

[bibr48-00131644221077135] Trepte S. Verbeet M. (2010). Allgemeinbildung in Deutschland—Erkenntnisse aus dem SPIEGEL Studentenpisa-Test. VS Verlag für Sozialwissenschaften.

[bibr49-00131644221077135] van der Flier H. Mellenbergh G. J. Adèr H. J. Wijn M. (1984). An iterative item bias detection method. Journal of Educational Measurement, 21, 131–145. 10.1111/j.1745-3984.1984.tb00225.x

[bibr50-00131644221077135] Vaughn B. K. Wang Q. (2010). DIF trees: Using classification trees to detect differential item functioning. Educational and Psychological Measurement, 70, 941–952. 10.1177/0013164410379326

[bibr51-00131644221077135] Wang W.-C. (2004). Effects of anchor item methods on the detection of differential item functioning within the family of Rasch models. Journal of Experimental Education, 72, 221–261. 10.3200/JEXE.72.3.221-261

[bibr52-00131644221077135] Wang W.-C. Shih C. L. Sun G. W. (2012). The DIF-free-then-DIF strategy for the assessment of differential item functioning. Educational and Psychological Measurement, 72, 687–708. 10.1177/0013164411426157

[bibr53-00131644221077135] Wang W.-C. Shih C.-L. Yang C.-C. (2009). The MIMIC method with scale purification for detecting differential item functioning. Educational and Psychological Measurement, 69, 713–731. 10.1177/0013164409332228

[bibr54-00131644221077135] Wang W.-C. Su Y.-H. (2004a). Effects of average signed area between two item characteristic curves and test purification procedures on the DIF detection via the Mantel-Haenszel method. Applied Measurement in Education, 17, 1–75. https://www.tandfonline.com/doi/pdf/10.1207/s15324818ame1702_2

[bibr55-00131644221077135] Wang W.-C. Su Y.-H. (2004b). Factors influencing the mantel and generalized Mantel-Haenszel methods for the assessment of differential item functioning in polytomous items. Applied Psychological Measurement, 28, 450–480. 10.1177/0146621604269792

[bibr56-00131644221077135] Wickelmaier F. Zeileis A. (2018). Using recursive partitioning to account for parameter heterogeneity in multinomial processing tree models. Behavior Research Methods, 50, 1217–1233. 10.3758/s13428-017-0937-z 28779459

[bibr57-00131644221077135] Wickham H. (2016). ggplot2: Elegant graphics for data analysis. Springer-Verlag New York. https://ggplot2.tidyverse.org/

[bibr58-00131644221077135] Zeileis A. Hothorn T. Hornik K. (2008). Model-based recursive partitioning. Journal of Computational and Graphical Statistics, 17, 492–514. 10.1198/106186008X319331

[bibr59-00131644221077135] Zwick R. (1990). When do item response function and Mantel-Haenzel definitions of Differential Item Functioning coincide. Journal of Educational and Behavioral Statistics, 15, 185–197. 10.3102/10769986015003185

[bibr60-00131644221077135] Zwick R. (2012). A review of ETS differential item functioning assessment procedures: Flagging rules, minimum sample size requirements, and criterion refinement. ETS Research Report Series, 2012, i–30. 10.1002/j.2333-8504.2012.tb02290.x

